# Recent developments in photoacoustic imaging and sensing for nondestructive testing and evaluation

**DOI:** 10.1186/s42492-021-00073-1

**Published:** 2021-03-19

**Authors:** Sung-Liang Chen, Chao Tian

**Affiliations:** 1 University of Michigan-Shanghai Jiao Tong University Joint Institute, Shanghai Jiao Tong University, 200240 Shanghai, China; 2 Engineering Research Center of Digital Medicine and Clinical Translation, Ministry of Education, 200030 Shanghai, China; 3 State Key Laboratory of Advanced Optical Communication Systems and Networks, Shanghai Jiao Tong University, 200240 Shanghai, China; 4 Department of Precision Machinery and Precision Instrumentation, University of Science and Technology of China, 230026 Hefei, Anhui China

**Keywords:** Photoacoustic imaging, Photoacoustic sensing, Nondestructive testing, Nondestructive evaluation, Photoacoustic microscopy

## Abstract

Photoacoustic (PA) imaging has been widely used in biomedical research and preclinical studies during the past two decades. It has also been explored for nondestructive testing and evaluation (NDT/E) and for industrial applications. This paper describes the basic principles of PA technology for NDT/E and its applications in recent years. PA technology for NDT/E includes the use of a modulated continuous-wave laser and a pulsed laser for PA wave excitation, PA-generated ultrasonic waves, and all-optical PA wave excitation and detection. PA technology for NDT/E has demonstrated broad applications, including the imaging of railway cracks and defects, the imaging of Li metal batteries, the measurements of the porosity and Young’s modulus, the detection of defects and damage in silicon wafers, and a visualization of underdrawings in paintings.

## Introduction

Nondestructive testing and evaluation (NDT/E) is the process of testing, examining, or evaluating materials, components, or assemblies for characterization without causing damage to the part or system [1]. The terms “nondestructive inspection” and “nondestructive examination” are also used to describe the same process as NDT/E. In contrast to destructive testing, which is used to determine the physical properties of materials such as their impact resistance, ductility, tensile strength, fracture toughness, and fatigue strength, NDT/E is more effective in noninvasively evaluating the characteristics of the materials. Therefore, NDT/E is a highly valuable technique in product evaluation and troubleshooting and has been widely used in fabrication and in-service inspections to ensure product integrity and reliability.

Currently, the most frequently used NDT/E techniques include liquid penetrant, magnetic particle, electromagnetic, visual, radiographic, and ultrasonic (US) testing [1]. Liquid penetrant testing is used to evaluate surface defects by checking residual penetrants in fissures and voids after applying penetrants to the materials. It can be conducted on magnetic and non-magnetic materials but does not work well on porous materials. Magnetic particle testing uses external magnetic fields to magnetize the piece under testing and then detect magnetic flux leakage caused by surface or subsurface discontinuities. However, this technique can only be applied to ferromagnetic materials. Electromagnetic testing induces electric currents, magnetic fields, or both inside a test object to observe the electromagnetic response caused by surface or subsurface defects. This method is limited to conductive materials [1]. Visual testing involves visual observation of a test object by human eyes or optical instruments to evaluate the presence of surface discontinuities. It is the most commonly used testing method in industry but suffers from limitations such as difficulty in identifying small defects and being vulnerable to surface conditions. Moreover, visual testing and the other three NDT/E techniques discussed above all suffer from the problem of the detection depth and can only detect surface or subsurface defects. By contrast, radiographic testing can be conducted inside a test object using radiating X-rays or gamma rays and yielding volumetric images of the object [1]. The major challenge for this technique is its safety because hazardous radiation is involved. US testing uses high-frequency sound waves to probe an object and analyzes echo signals to characterize the test object. US testing can be used to see deep inside the object and does not incur safety problems. However, this technique typically has a poor axial resolution owing to the limited bandwidth of ultrasound transducers [2] and is difficult to apply to rough, irregular, and nonhomogeneous objects.

Photoacoustic (PA), or optoacoustic, technology-based NDT/E is an alternative to the aforementioned NDT/E techniques and has attracted considerable attention over the years. The technique is based on the PA effect, which was discovered by Alexander Graham Bell in 1880, and indicates the fact that varying light intensity can produce sound waves [3]. The PA effect was explored for a spectroscopic analysis of gases in the early 20th century by Vengerov at the State Optical Institute, Leningrad [4, 5]. Its applications in solids and for NDT/E were not started until the 1970 s. In 1973, Rosencwaig first extended the PA effect from gas to solids and obtained valuable information about the optical properties of solids and biological materials, such as Cr_2_O_3_ powder and hemoglobin [6, 7]. In 1978, Wong et al. [8] demonstrated that the technique can be applied to study the microstructures of solid surfaces, that is, for a nondestructive evaluation of surface flaws and subsurface inhomogeneities. Since then, PA technology has proven to be a deep-penetrating, high-resolution, three-dimensional (3D) NDT/E tool and has been applied in a range of testing fields, such as the damage evaluation of carbon fiber-reinforced plastic (CFRP) composites [9], measurement of weld defects [10], and visualization of dendrite growth in Li metal batteries [11], to name just a few.

PA imaging is a hybrid imaging modality that visualizes optical absorption contrast through the PA effect [12, 13], a physical phenomenon that converts absorbed light into sound (optical energy to acoustic energy) owing to thermoelastic expansion. A pulsed laser is typically used to illuminate a sample for an efficient generation of PA waves, which are basically the same as US waves. Only the region of the sample that can absorb the laser energy will produce PA waves, while the other region of the sample that does not absorb laser energy will not generate PA waves. This is called the optical absorption contrast. These PA waves are finally detected by ultrasound transducers, and an image formation algorithm may need to be further used to reconstruct the absorption map in the sample. The above procedure is called PA imaging. Currently, researchers use either a commercial PA imaging system or a home-built system to conduct PA imaging.

Laser ultrasonics is a special approach to PA technology for NDT/E and has achieved success in composite inspections in the industry and on-line hot tube thickness measurements for the metallurgical field [2, 14]. The uniqueness of the laser ultrasonics approach lies in its ultrasound detection strategies. Instead of using microphones or piezoelectric transducers, laser ultrasonics typically employs an optical interferometer for the remote detection of laser-induced US vibrations [2, 14]. However, ultrasound detection by optical interferometers typically suffers from the problem of low sensitivity [14] and is more costly and complex to use. As a result, laser ultrasonics can only be used in specific applications. Recent work on laser ultrasonics will also be described in this paper.

This paper is organized as follows. The background of NDT/E is introduced in Introduction secion. The PA wave equation and solution are reviewed in PA wave equation and solution section. PA technologies for NDT/E are described in PA technologies section in detail. Then, recent developments in PA technology-based NDT/E for different materials and broad applications are presented in Applications in various materials section. Finally, the conclusions and outlooks are given.

## Photoacoustics for NDT/E

### PA wave equation and solution

The generation and propagation of PA signals *p*(**r**, t) under the condition of thermal confinement in a lossless homogenous medium is governed by the following PA wave equation:

1 $${\nabla ^2}p(\mathbf{r},t) - \frac{1}{{{c^2}}}\frac{{{\partial ^2}}}{{\partial {t^2}}}p(\mathbf{r},t)= - \frac{\beta }{{{C_p}}}\frac{\partial }{{\partial t}}H(\mathbf{r},t),$$

where *H*(**r**, t) is the heating source, representing the energy deposited in the tissue per unit volume per unit time; *c* is the speed of sound; *β* is the isobaric thermal volume expansion coefficient; and *C*_*p*_ denotes the specific heat capacity at a constant pressure. Under the condition of stress confinement, the duration of the laser pulse is much shorter than the time it takes for sound to travel across the heated region. The heating function can be decomposed as *H*(**r**, *t*) ≈ *A*(**r**)*δ*(*t*), where *A*(**r**) is the energy deposited in the tissue per unit volume and *δ*(*t*) is the Dirac delta function. As a result, the initial acoustic pressure *p*_0_(**r**) at position **r** can be written as follows:

2 $${p_0}(\mathbf{r})=\Gamma A(\mathbf{r}),$$

where Γ is the Grueneisen coefficient. By solving Eq. (1) using Green’s function, the acoustic pressure *p*(**r**, *t*) recorded by a detector at location **r**_d_ can be written as follows:

3 $$p({\mathbf{r}_{\text{d}}},t)=\frac{1}{{4\pi {c^2}}}\frac{\partial }{{\partial t}}\int_{V} {\frac{{{p_0}({\mathbf{r}_{\text{s}}})}}{{|{\mathbf{r}_{\text{s}}} - {\mathbf{r}_{\text{d}}}|}}\delta \left( {t - \frac{{|{\mathbf{r}_{\text{s}}} - {\mathbf{r}_{\text{d}}}|}}{c}} \right)dV} ,$$

where d*V* is a 3D volume element at **r**_s_. This equation mathematically represents the spherical Radon transform and suggests that the initial acoustic pressure *p*_0_(**r**_s_) in a small tissue volume at **r**_s_ linearly contributes to the recorded acoustic pressure *p*(**r**_d_, *t*).

### PA technologies

A variety of PA technologies have been employed in NDT/E. According to the approaches for PA wave excitation, light-sample interactions, and PA wave detection, the technologies can be categorized into a few types, as detailed in the following.

#### NDT/E with modulated CW laser for PA wave excitation

**Fig. 1:**
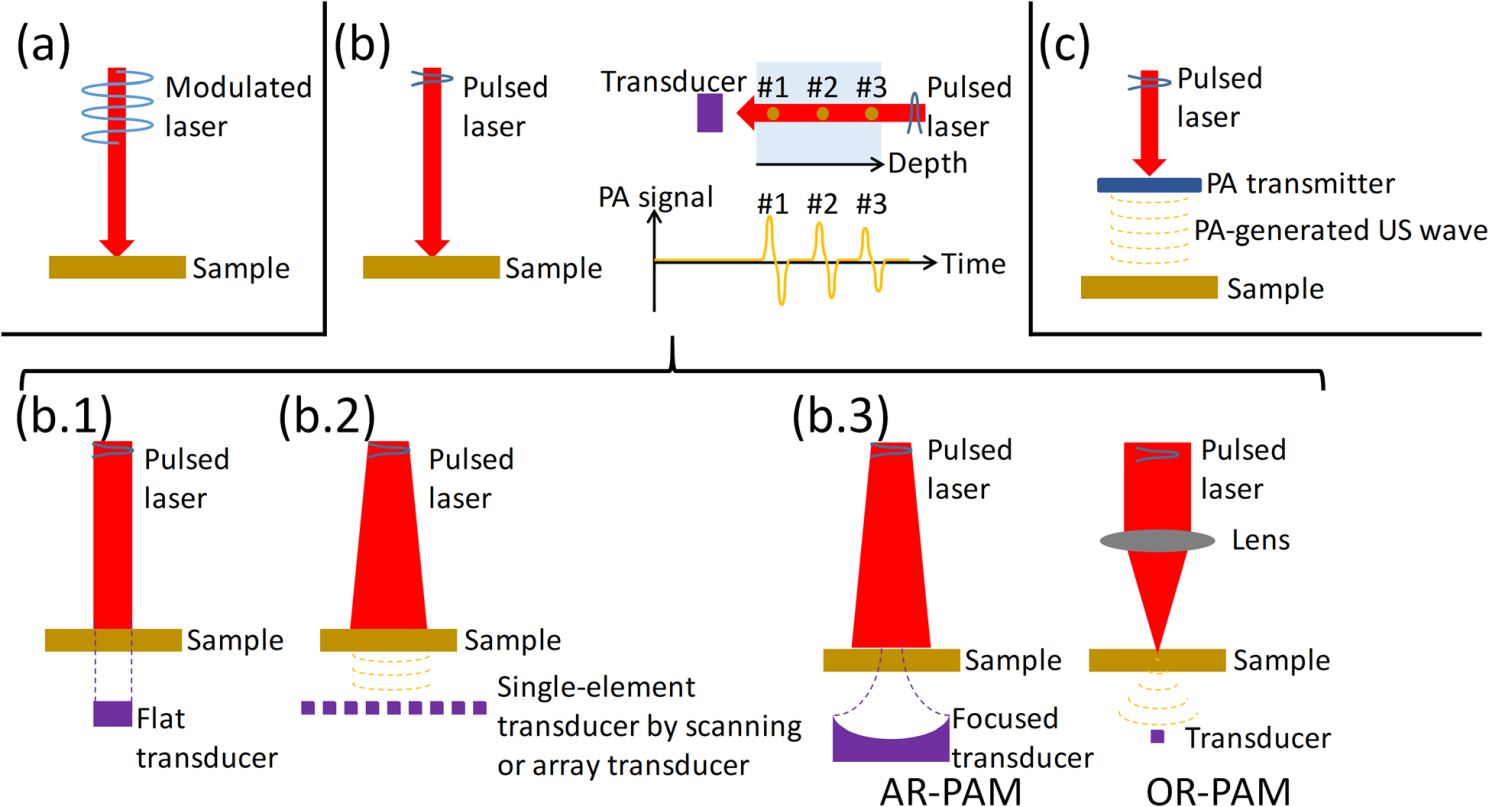
PA technologies for NDT/E. **a**: NDT/E with modulated CW laser for PA wave excitation (NDT/E with modulated CW laser for PA wave excitation section); (**b**): NDT/E with pulsed laser for PA wave excitation (NDT/E with pulsed laser for PA wave excitation section) (left) and illustration of the PA A-line signal (right). A flat or focused piezoelectric transducer can be used. (b.1): PA A-line and simple imaging; (b.2): PACT; and (b.3): AR-PAM (left) and OR-PAM (right). In (**b**), transmission mode is plotted for easy illustration. Note that OR-PAM in (**b**.3) can also be realized using a modulated CW laser; (**c_**: NDT/E with PA-generated US waves (NDT/E with PA-generated US waves section)

The scheme has been extensively used for NDT/E [10, 15–25]. A schematic is shown in Fig. 1a. A continuous-wave (CW) laser was used as the light source. The light intensity of the laser is modulated using an optical chopper (mechanically) or a function generator (electronic). The optical chopper or function generator can be used to control the modulation frequency. The modulated CW laser beam is used to illuminate the sample (which can be a solid, liquid, or gas), and the excited PA wave is typically detected using a piezoelectric transducer. The detected PA signals can be further amplified using a lock-in amplifier. PA signal amplitudes are typically used to provide useful information, and for certain applications, two-dimensional (2D) scanning of the focused laser beam over the sample may be conducted to produce different images. Different wavelengths of the laser beam can be used to produce a PA spectrum, which provides information on the absorbing components of the sample, which is a process called PA spectroscopy. In addition, using a gas-filled chamber (also called a PA cell), PA signals can be further amplified by adjusting the modulation frequency to match the resonance of the PA cell.

#### NDT/E with pulsed laser for PA wave excitation

Compared with a CW laser, the pulsed laser is able to generate short US pulses (Fig. 1b). As shown in Fig. 1b, without a loss of generality, assume that the three absorbers are located at different depth positions. When the absorbers are illuminated by a pulsed laser, PA signals from the three absorbers are generated and detected using an ultrasound transducer. Because of the different time of flight of the PA signals from the three absorbers to the ultrasound detector, the PA signals will appear at different time positions (Fig. 1b), corresponding to a one-dimensional (1D) image (or 1D profile) along the depth direction, which is called a PA A-line signal. The shorter the laser pulse duration is, the shorter the generated US pulses, and the broader the bandwidth of the PA A-line signals [26]. The laser pulse duration is typically a few nanoseconds, and the generated PA A-line signals have bandwidths of several to tens of megahertz. A flat or focused piezoelectric transducer was used. By scanning the transducer (or the sample), PA images can be obtained. Because one laser excitation produces the depth profile, as mentioned above, 1D scanning enables a 2D image (B-scan), and 2D scanning generates a 3D image. The axial resolution is mainly limited by the bandwidth of the transducer. The lateral resolution depends on the different configurations of the PA imaging setup, as elaborated in the following.

1. PA A-line and simple imaging. As shown in Fig. 1 (b.1), the sample was illuminated using a pulsed laser. A flat transducer is usually applied. Scanning can be applied to render a PA image. Here, “simple imaging” mainly refers to imaging with an unfocused pulsed laser beam (for excitation) and an unfocused transducer (for detection). The lateral resolution is determined by the aperture of the transducer, which is on the order of millimeters. In this scheme, PA signal amplitudes and spectra, depth profiles, and B-mode images may provide useful information [27–37].
2. PA computed tomography (PACT). To enhance the spatial resolution, an image reconstruction algorithm can be further employed. A schematic is shown in Fig. 1 (b.2). The laser beam is usually expanded to cover the ROI. At one burst of the laser pulse, PA signals originating within the ROI are detected by transducers placed at different positions. This can be realized by scanning a single-element transducer or using an array transducer. Then, PA images can be reconstructed using different algorithms, such as delay-and-sum and back-projection methods. Overall, transducers with a high frequency, broad bandwidth, and wide receiving angle are desired to provide a high spatial resolution, which is typically hundreds of micrometers. In addition, using the array transducer, point-by-point scanning can be avoided, and thus, the image acquisition time can be significantly shortened to realize real-time imaging with a fast reconstruction time. In this scheme, PA images are mainly used for NDT/E [38, 39].
3. PA microscopy (PAM). The spatial resolution can be further boosted using PAM, which can be further categorized into acoustic-resolution PAM (AR-PAM) and optical-resolution PAM (OR-PAM), as shown in Fig. 1 (b.3). In AR-PAM, the laser beam illuminates the sample, and a single-element focused transducer is used [40]. The laser beam spot size is larger than the acoustic focal spot size of the focused transducer. Therefore, the lateral resolution is determined by the focusing ability of the focused transducer and is on the order of tens to hundreds of micrometers. In OR-PAM, the laser beam is strongly focused on the sample to excite PA signals [9, 11, 41–45]. A high lateral resolution is thus enabled by the focused laser beam spot size and can be up to a few micrometers and even submicrons. The high-resolution of OR-PAM renders it suitable for applications where high-resolution visualization is essential. However, compared with PACT using an array transducer, PAM requires point-by-point scanning and thus takes more time for image acquisition. Similar to PACT, in PAM, 2D or 3D images are mainly utilized to provide potential information for a nondestructive investigation.

**Fig. 2:**
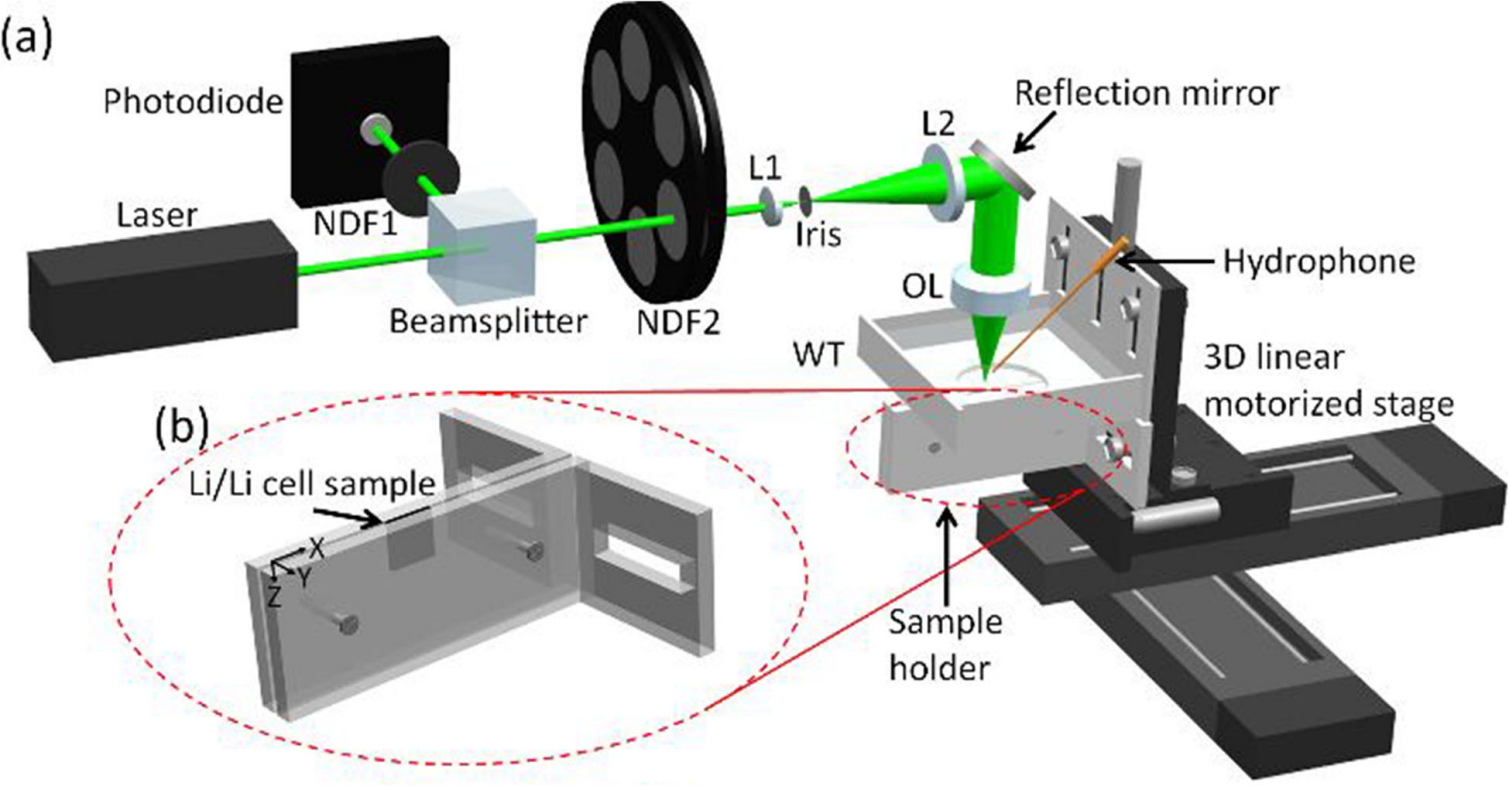
Typical PA imaging setup for NDT/E [11]. (**a**): Schematic of OR-PAM imaging setup; (**b**): Custom-made sample holder. NDF1: neutral density filter 1; NDF2: neutral density filter 2; L1: lens 1; L2: lens 2; OL: objective lens; WT: water tank

A typical OR-PAM imaging setup is shown in Fig. 2 [11]. The setup was used to visualize the Li metal batteries. A pulsed laser was used to excite the PA signals. As shown in Fig. 2(a), the laser emitted from the laser head was split into two beams: one detected by a photodiode and used as trigger signals, and another spatially filtered and then focused by an objective lens and used for PA wave excitation. Figure 2(b) shows a custom-made sample holder that facilitates the PA imaging procedure. A water tank was used to ensure acoustic coupling from the sample to the hydrophone, which was immersed in water. The 3D linear motorized stage was used to scan the sample during image acquisition. Finally, an OR-PAM image can be obtained.

#### NDT/E with PA-generated US waves

Apart from the PA waves generated by laser illumination on the sample, PA-generated US waves can also be utilized to interrogate the sample for NDT/E [46–51]. A schematic is shown in Fig. 1(c). The materials used for PA wave generation are termed PA transmitters. Compared with piezoelectric transducers, the PA approach is a natural solution for generating broadband US pulses. Although a previously low PA conversion efficiency was an issue and thus limited its practical uses, owing to the advancement of fabrication technologies and material sciences, PA transmitters with an enhanced PA conversion efficiency have recently been developed [52]. For efficient PA wave generation, PA transmitters with high absorption coefficients and high thermal expansion coefficients are desired. For broadband PA wave generation, a laser with a short pulse duration for illumination and a PA transmitter with a thin effective thickness for absorption have to be employed. As another advantage, PA transmitters can be tiny and integrated with optical fibers, which facilitate selected applications such as an endoscopic investigation of regions that are difficult to access with piezoelectric transducers. Note that the PA-generated US waves (after or without interrogating the sample) can be detected using an ultrasound transducer [46, 49, 51] or using optical ultrasound detection technology [47, 48, 50].

#### NDT/E with all‐optical PA wave excitation and detection

Optical US detection is an alternative technique to piezoelectric transducers. Compared with piezoelectric transducers, optical US detection generally has the advantages of a broader bandwidth, higher spatial resolution (tiny active detection area), wider acceptance angle, and immunity to electromagnetic interference [53]. A fiber-optic hydrophone was demonstrated, but its sensitivity was significantly lower than that of piezoelectric transducers [54]. Optical resonators or interferometers have recently been used for optical US detection, and their sensitivity has been significantly enhanced. For example, a record-low NEP of < 10 Pa was reported in optical US detectors using plano-concave optical microresonators [55]. Another figure of merit is defined as the US detection sensitivity divided by the detector size, which is called the sensitivity density. Compared with piezoelectric transducers, optical US detection has a much better performance in terms of sensitivity density and can be useful for high-resolution detection and applications [31]. Further, some optical US detection techniques enable noncontact remote sensing of US waves [10, 19, 31, 35, 36, 48], which is particularly useful in certain NDT/E applications. A summary of the PA technologies used for NDT/E is provided in Table 1.

**Table 1:** Summary of PA technologies used for NDT/E

PA technology for NDT/E	Lateral resolution^a^	Advantage	Ref.
1. Modulated CW laser for PA wave excitation	Approximately micron	Cost-effective light sources	[10, 15–25]
2. Pulsed laser for PA wave excitation			
2.1 PA Aline and simple imaging	Approximately millimeter	Useful information in PA Aline	[27–37]
2.2 PACT	Hundreds of micron		[38, 39]
2.3 PAM	Tens of micron (AR-PAM); approximately micron (OR-PAM)	High lateral resolution	[9, 11, 40–45]
3. PA-generated US waves as sources	Approximately micron	Broad bandwidth for generated US waves; miniature size of PA transmitters	[46–51]
4. All-optical PA wave excitation and detection	Approximately micron	Broad bandwidth, wide acceptance angle, and noncontact for PA wave detection	[10, 19, 31, 34, 35, 48]

^a^The highest lateral resolution that has been demonstrated

A summary of the technical specifications of PA technologies used for NDT/E (representative examples) is provided in Table 2. As can be seen, laser wavelengths of 532 and 1064 nm and ultrasound transducers with frequencies of tens of megahertz are commonly used.

**Table 2:** Technical specifications of PA technologies used for NDT/E (representative examples)

PA technology for NDT/E	Laser wavelength (nm)^a^	Lateral resolution; axial resolution (µm)	US frequency of ultrasound transducer (MHz)	Ref.
1. Modulated CW laser for PA wave excitation	830	100; NA	NA	[19]
2. Pulsed laser for PA wave excitation				
2.1 PA Aline and simple imaging	1064	NA; NA	1−100	[37]
2.2 PACT	532	2000; NA	40	[38]
2.3 PAM^b^	1064	525; NA	20	[40]
	532	3.3; 26	35	[11]
3. PA-generated US waves as sources	532	1.7^c^; NA	20 and 93	[51]
4. All-optical PA wave excitation and detection	532	NA; NA	NA	[35]

^a^For PA excitation; ^b^For AR-PAM [40] and OR-PAM [11]; ^c^For the transmitter. NA: Not applicable

### Applications in various materials

#### Metal

In this subsection, earlier studies on a preliminary investigation into NDT/E on metallic films and plates, as well as their detection of cracks and defects, are first introduced. Several recent applications are then described.

**Fig. 3:**
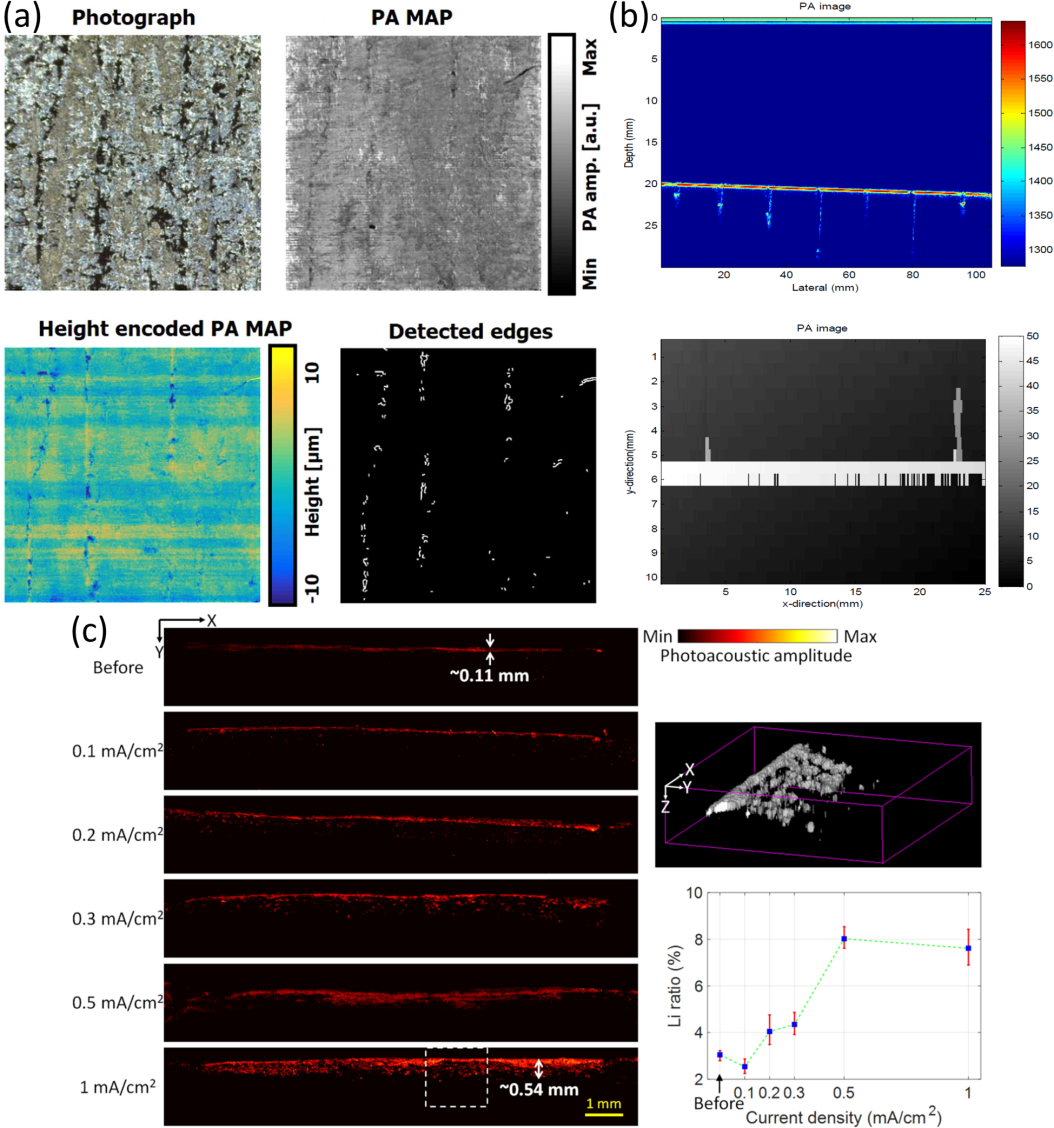
Representative applications of metal using PA technologies for NDT/E. (**a**): Detected edge of the seam crack surface by OR-PAM images [42]; (**b**): PA images of high-speed rail surface cracks: rail head (top) and rail web (bottom) [38]; (**c**): Li deposition of Li/Li cell samples imaged using OR-PAM. Li metal electrode of Li/Li cell samples before and after charging at different current densities (left), representative 3D visualization of the case of 1 mA/cm^2^ (top-right), and quantitative analysis of Li deposition (bottom-right) [11]

The first part can be further categorized into three types: (1) Metallic films and plates. Veith [41] used a focused laser beam to conduct PAM imaging (lateral resolution of 5 μm) of a Au film. Pelivanov et al. [30] conducted a theoretical analysis of the PA conversion of a metal film deposited on a transparent substrate, which implies a method for the NDT/E of submicron metal coating properties. (2) Surface cracks and defects. Grégoire et al. [18] found a nonlinear process of PA generation (frequency mixing observed in the PA spectra) owing to the presence of cracks, which can be utilized for crack detection. Zakrzewski et al. [20] showed nonlinear PA imaging of surface breaking cracks on a metallic plate. Jeon et al. [42] employed OR-PAM to detect metal surface defects (unclassified and seam cracks) in metal plates [Fig. 3(a)]. Furthermore, by utilizing the depth-resolving ability of OR-PAM, crack edges can be extracted. (3) Internal cracks and defects. Oe et al. [19] demonstrated a self-coupling sensor to detect very small internal defects (0.07 mm). Shiraishi et al. [22] used PAM to visualize an internal defect in the welded region of an Al plate. In addition, the size of the internal defect can be measured using PAM imaging. Kato et al. [10] further demonstrated the PAM imaging of surface cracks and internal defects in the welded region of an Al plate. It is worth mentioning that PAM can also image wedge-typed subsurface defects [17].

For the second part, thus far, there have been specific applications reported: (1) Railway defects and cracks. Yan et al. [23] utilized the PA technique to demonstrate nondestructive imaging of cracks in Chinese national standard railway steel samples. In addition, Sun et al. [38, 39] conducted experiments to obtain PA reconstructed images of high-speed rail surface defects [Fig. (3b)]. Some damage information of the rail defect (e.g., appearance and depth of the defect) can be identified. (2) Steel rebar corrosion monitoring. Zou et al. [49] used PA-generated US waves for nondestructive corrosion detection of steel reinforced rebar samples with different corrosion rates. The method has several advantages, including a miniature device size, noncontact approach, and high spatial resolution. Du et al. [50] further designed and fabricated an all-optical device consisting of distributed PA transmitters and FBG US sensors for steel rebar corrosion monitoring. (3) Imaging of Li metal batteries. Liu et al. [11] utilized OR-PAM imaging to visualize Li protrusions in Li metal batteries, aiming to overcome the Li dendrite problem Fig. 3(c)]. In addition, quantification of the deposited Li mass by processing OR-PAM images was also demonstrated [45]. In the future, real-time PAM imaging of Li dendrites in Li metal batteries can be useful for *in situ* and *operando* observations of Li metal batteries during cycling, which provides insight into the fundamental mechanisms of Li dendrite growth. (4) Other (optical reflection coefficient and heavy metal contaminants). The optical reflection coefficient of the metal mirrors was measured using a PA cell [24]. Liu et al. [25] also utilized a PA cell to evaluate soil heavy metal contaminants.

#### Composite

Karabutov et al. [27] studied layered graphite-epoxy composites with different porosities using the pulsed PA method. In addition, the defects and their depth in the graphite epoxy composites can also be determined by conducting a PA spectral and correlation analysis [29].

Podymova et al. [33] demonstrated the measurement of phase velocities, which can be utilized to determine the local porosity of SiC-particle-reinforced silumin-matrix composites. The local Young’s modulus of the composites can be determined by the pulsed PA method, and the effect of the porosity on the local Young’s modulus was quantitatively evaluated [36].

**Fig. 4:**
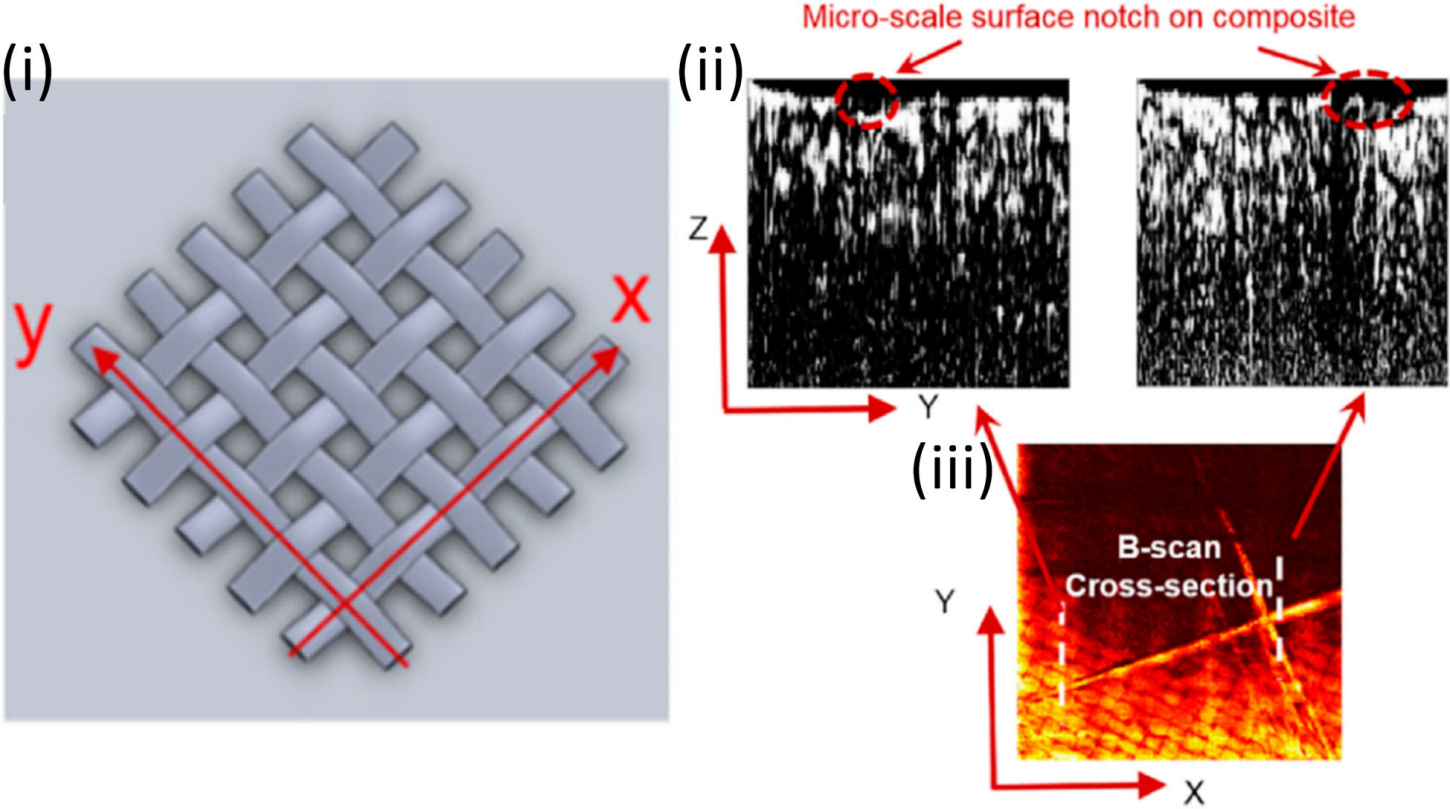
One representative application of composite using PA technologies for NDT/E: Detection of damage in CFRP composites [9]. (**a**)**:** Illustrative image of grid pattern; (**b**): B-scan images; and (**c**)**:** C-scan image (bottom). The B-scan images are along the white lines in the C-scan image

Wang et al. [43, 44] detected damage precursors in CFRP composites using OR-PAM imaging with a high spatial resolution. Multi-scale damage detection (e.g., surface notches at the microscopic scale and delamination on the macroscopic scale) of CFRP composites through OR-PAM imaging has recently been further demonstrated (Fig. 4) [9].

#### Silicon

Berquez et al. [16] detected defects at different depths in silicon wafers by varying the modulation frequency of the laser source (3.9 kHz to 1 MHz).

Kozhushko and Hess [48] used a PA transmitter, which is a stainless-steel spherical mirror, to produce PA-generated focused US pulses with a broad bandwidth (> 100 MHz). The US pulses launched in the sample, and the sensitive detection of mechanical discontinuities, such as an edge in a silicon wafer, were demonstrated by observing the transient elastic disturbance at the sample surface, which was probed using a laser beam.

**Fig. 5:**
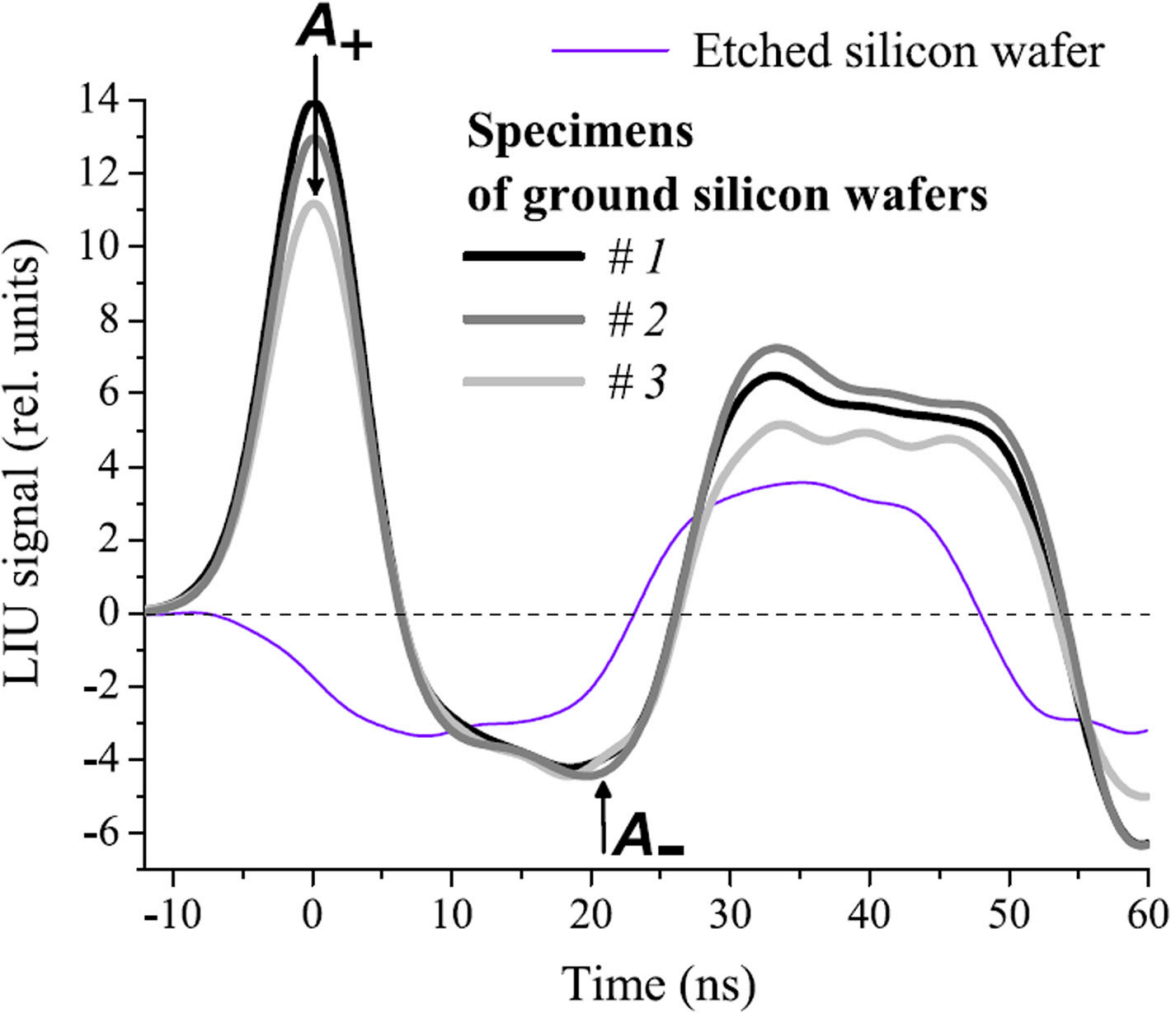
One representative application of silicon using PA technologies for NDT/E: PA A-line signals in ground silicon wafers with different damage depth [37]. The measured values of *A*_+_ and *A*_−_ can be used to determine the subsurface damage depth. LIU signal, laser-induced US signals (i.e., PA signals)

Podymova and Karabutov [32] demonstrated the measurement of the thickness of the damaged layer in machined silicon wafers using the pulsed PA method. Single-crystal silicon wafers were also evaluated using PA technology [37]. The silicon wafers were illuminated using a pulsed laser, and PA A-line signals were generated owing to different mechanisms between undamaged and damaged silicon layers, i.e., a concentration-deformation mechanism for the undamaged silicon layer and a thermoelastic mechanism for the damaged layer. The different mechanisms are attributed to the different lifetimes of the photoexcited carriers, which is much shorter for the latter mechanism (thermoelastic). PA A-line signals were observed from four samples (Fig. 5), i.e., an etched silicon wafer (undamaged) and three specimens of ground silicon wafers with damaged layers, the subsurface damage depth of which was determined using scanning electron microscopy [37]. As can be seen, in contrast to the three damaged specimens, the undamaged silicon wafer shows a negative plateau first, which corresponds to an increase in the concentration of photoexcited carriers. By contrast, the three damaged specimens show a positive peak first (a compression phase) followed by a negative plateau (a rarefaction phase), which correspond to the thermoelastic part (the damaged layer) and the concentration-deformation part (the undamaged layer beneath the damaged layer), respectively. Empirically, the ratio of the amplitudes of the compression and rarefaction phases (*A*_+_ and *A*_−_ in Fig. 5, respectively) has a linear dependence on the subsurface damage depth, and thus, this technology can be used to determine the subsurface damage depth in ground silicon wafers.

#### Others

**Fig. 6:**
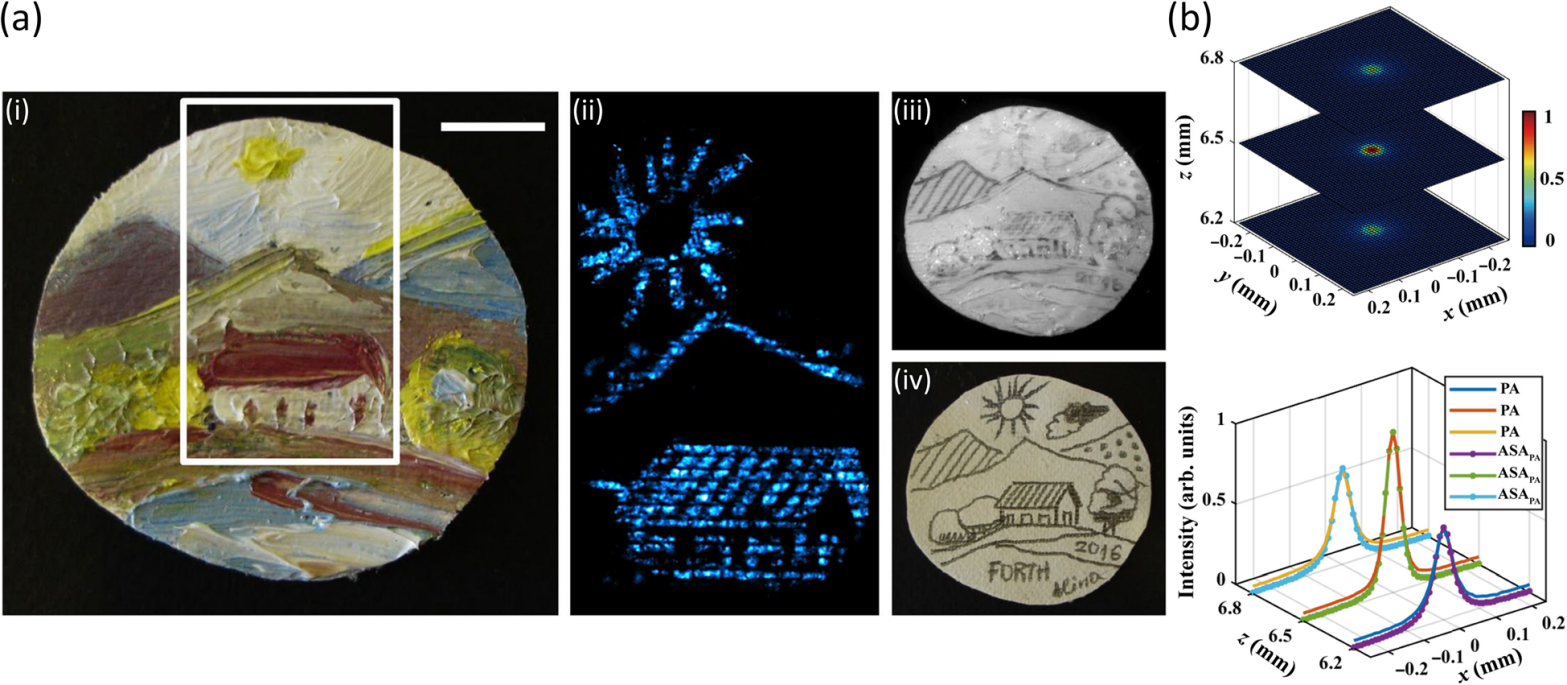
Representative applications of other materials using PA technologies for NDT/E. (**a**): PA detection of underdrawings in paintings [40]. (i) Brightfield view of the painting (rural landscape), (ii) PA image of the underlying sketch of the painting over a region indicated by the white box in (i), (iii) NIR image of the underdrawing at 1200 nm, and (iv) Brightfield view of the original pencil sketch before overpainting. Scale bar = 1 cm; (**b**): SIR measurement of ultrasound transducers using PA method [51]. 2D acoustic intensity profile of a 93 MHz focused transducer on *x*-*y* plane at *z* = 6.2, 6.5, and 6.8 mm (top). Comparison of the PA-measured and calculated results (bottom), showing good agreement. The PA-measured results mean the acoustic intensity distribution measured by the PA method (denoted as “PA” in the legend); the calculated results mean the acoustic intensity distribution calculated by the angular spectrum approach (ASA) based on PA-measured SIR (denoted as “ASA_PA_” in the legend)

Kim and Netzelmann [15] employed PAM imaging (using a laser modulation frequency of 33.8 kHz) and high-frequency US imaging (80 MHz) to detect delaminated regions of ceramic coatings (approximately 300 μm thickness) on steel substrates. Ségur et al. [31] developed a picosecond pump-probe technique to measure the transverse elastic properties and the complex refractive index (at a wavelength of 796 nm) of a single carbon fiber with a mean diameter of 10 μm. In addition, Tserevelakis et al. [40] utilized PA imaging with a resolution of 525 μm to reveal the underdrawings in paintings. A nanosecond pulsed laser at 1064 nm was used to illuminate an oil painting on canvas from its reverse side, and the hidden pencil sketch lines were clearly detected [Fig. 6(a)]. Lu et al. [51] utilized PA-generated US waves to produce a point-like source with a broad bandwidth and small size, which enables a high-precision (resolution of 1.7 μm) acoustic field measurement. A measurement of the spatial impulse response (SIR) of ultra-high-frequency ultrasound transducers was demonstrated [Fig. 6(b)].

A summary of the materials and applications of PA technologies for NDT/E is provided in Table 3.

**Table 3:** Summary of the materials and applications by PA technologies for NDT/E

Material	Application; detection	Technology^a^	Ref.
Metal	General cracks and defects	1; 2.1; 2.3	[10, 17–20, 22, 30–32]
	Railway cracks and defects	2.1; 2.2	[23, 38, 39]
	Steel rebar corrosion monitoring	3	[49, 50]
	Li metal battery	2.3	[11, 45]
	Optical reflection coefficient; heavy metal contaminants	1	[24, 25]
Composite	Defects	2.1	[29]
	Porosity	2.1	[27, 33]
	Young’s modulus	2.1	[36]
	Damage	2.3	[9, 43, 44]
Silicon	Defects	1; 3	[16, 48]
	Damage	2.1	[32, 37]
Others	Coatings	2.1	[15]
	Elastic properties and complex refractive index	2.1	[31]
	Underdrawings in paintings	2.3	[40]
	Precision acoustic field measurement	3	[51]

^a^See “PA technology for NDT/E” in Table 1

## Conclusions and outlooks

Several PA technologies have been employed for NDT/E, such as the use of a modulated CW laser or a pulsed laser to excite PA signals, PA-generated US waves to interrogate samples, and an all-optical scheme to realize non-contact remote sensing. Either the interaction of light and the samples, or the interaction of PA-generated US waves and samples, can be considered in NDT/E. Depending on the materials to be characterized or imaged and their environments, a suitable PA technology for specific NDT/E applications may be chosen. For NDT/E using PA technology, a variety of materials have been studied. Applications range widely in different fields such as the semiconductor industry (e.g., defects and damage in silicon wafers), mass transport (e.g., railway cracks and defects), and the energy industry (e.g., imaging of Li metal batteries).

PA technology has proven to be effective for NDT/E. PAM possesses a high spatial resolution, particularly OR-PAM, which is useful for visualizing fine structures. Meanwhile, fast and real-time PAM imaging systems can facilitate extended studies. By contrast, PA-generated US waves naturally enable broadband US emissions, opening up new opportunities for NDT/E. With the advancement of PA technologies in different aspects (e.g., detection sensitivity, imaging speed, and spatial resolution), the challenges previously encountered in NDT/E applications are likely to be overcome, and more NDT/E applications by taking advantage of the advanced PA technologies may become possible.

Compared with US imaging used in NDT/E, PA imaging offers three advantages. (1) PA imaging can provide a high spatial resolution using the OR-PAM configuration, which can easily realize a microscale resolution. (2) PA imaging provides information associated with optical absorption contrast, which can be useful for certain applications. (3) Multiple wavelengths for PA wave excitation can be used to perform multispectral PA imaging, which helps identify different materials featuring distinct absorption spectra and optimizing imaging effects.

The limitations of PA technology for NDT/E are discussed. (1) A major limitation is the penetration depth of PA technology. For biomedical imaging applications, compared with pure optical microscopy, PA imaging can break the optical diffusion limit (transparent mean free path of approximately 1 mm in the skin) and enable a high US resolution at an imaging depth of up to a few centimeters in biological tissue [56]. However, for NDT/E applications using PA technology, the samples are typically not as transparent as biological tissue. For example, visible light can penetrate slightly into materials such as metal and silicon, not to mention the light scattering (or diffusion) inside them. Therefore, unlike US imaging for NDT/E, PA technology used to interrogate the samples is typically limited to a shallow depth from the sample surface. The use of the light wavelength for PA excitation with a deeper penetration may provide a solution, for example, using a light wavelength of 1550 nm for silicon. (2) Another limitation of PA technology for NDT/E is a contact operation using piezoelectric transducers. Currently, piezoelectric transducers are the most commonly used in PA imaging and sensing, and require acoustic coupling agents applied between the sample and ultrasound transducer. Researchers either use US gel or water as acoustic coupling agents. However, these methods are not ideal for industrial applications. Applying US gel or water generally causes inconvenience and even infeasibility in certain NDT/E applications. In addition, in US imaging for NDT/E, an ultrasound transducer is used for transmitting and receiving US waves such that it is usually in direct contact with the sample. By contrast, in PA technology for NDT/E, the transducer is opaque and a certain working distance between the transducer and the sample is needed to avoid blocking light for the sample illumination. This makes the optimized arrangement of the light illumination part, the acoustic detection part, and the acoustic coupling agents more complicated using PA technology than the US counterpart for NDT/E. As stated in NDT/E with all-optical PA wave excitation and detection section, all-optical and/or non-contact approaches have the potential to overcome the above challenges [53, 56, 57]. (3) Device cost is another limitation of PA technology. In contrast to US imaging, light illumination is also a key aspect of PA technology. Nanosecond pulsed lasers are typically expensive, particularly for wavelength-tunable lasers (OPO or dye lasers). Commercial PA imaging systems can cost approximately one million dollars [58]. Alternatively, low-cost light-emitting diodes (LEDs) are being explored as a substitute light source in PA systems [59]. Commercial LED-based PA imaging systems (Acoustic X, Cyberdyne Inc., Japan) are currently available, although the imaging performance is still limited compared with laser-based PA imaging systems.

Despite the current limitations, there are potential future directions of PA technology for NDT/E. Future directions include, but are not limited to, combined PA and US imaging for NDT/E, structural health monitoring (SHM), and the microscopy of metal: (1) Because piezoelectric transducers are typically used in PA systems, transducers can also be used for US imaging. Therefore, it is natural and usually seamless to integrate the two imaging modalities. The dual-modality approach is advantageous in providing complementary information, a scalable resolution, and the imaging depth. PA technology will be a good supplement for applications where US imaging has been extensively used, such as studying the formation of cracks. (2) SHM detects damage and the performance of mechanical structures (e.g., bridges, pressure containers, and aircraft) to monitor their safe operation. For example, SHM used for aerospace structures can greatly improve the level of safety and reduce expenses for maintenance and repair. Another example is monitoring railway cracks, as described in Metal section. Acoustic methods including pulse echo and transmission within the acoustic frequency range of 100 kHz to 1 MHz has been commonly used for SHM. PA technology may also find practical applications in SHM. (3) PAM enables a high resolution, and metal strongly absorbs light. Therefore, PA technology is useful for observing metal microstructures with high resolution and high contrast. For example, as stated in Metal section, PA technology has been used to study the Li dendrite problem [11, 45]. Other related applications are also worth exploring.

## Data Availability

Not applicable.

## Abbreviations

NDT/E: Nondestructive testing and evaluation
US: Ultrasonic
PA: Photoacoustic
3D: Three-dimensional
CFRP: Carbon fiber-reinforced plastic
CW: Continuous-wave
2D: Two-dimensional
1D: One-dimensional
PACT: Photoacoustic computed tomography
PAM: Photoacoustic microscopy
AR-PAM: Acoustic-resolution photoacoustic microscopy
OR-PAM: Optical-resolution photoacoustic microscopy
LED: Light-emitting diode
SHM: Structural health monitoring
ASA: Angular spectrum approach
