# Correction to: Recent developments in photoacoustic imaging and sensing for nondestructive testing and evaluation

**DOI:** 10.1186/s42492-021-00077-x

**Published:** 2021-04-29

**Authors:** Sung-Liang Chen, Chao Tian

**Affiliations:** 1 University of Michigan-Shanghai Jiao Tong University Joint Institute, Shanghai Jiao Tong University, Shanghai, 200240 China; 2 Engineering Research Center of Digital Medicine and Clinical Translation, Ministry of Education, Shanghai, 200030 China; 3 State Key Laboratory of Advanced Optical Communication Systems and Networks, Shanghai Jiao Tong University, Shanghai, 200240 China; 4 Department of Precision Machinery and Precision Instrumentation, University of Science and Technology of China, Hefei, 230026 Anhui China

**Correction to: Vis Comput Ind Biomed Art 4, 6 (2021)**

**https://doi.org/10.1186/s42492-021-00073-1**

Following publication of the original article [1], the authors identified an error in the article title. The first word ‘Review’ is added mistakenly by the typesetter.

The incorrect article title is: Review recent developments in photoacoustic imaging and sensing for nondestructive testing and evaluation.

The correct article title is: Recent developments in photoacoustic imaging and sensing for nondestructive testing and evaluation.

The article title has been updated in this correction and the original article [1] has been corrected.
